# *Megasphaera elsdenii* and *Saccharomyces Cerevisiae* as direct fed microbials during an in vitro acute ruminal acidosis challenge

**DOI:** 10.1038/s41598-022-11959-2

**Published:** 2022-05-13

**Authors:** Hugo F. Monteiro, Bruna C. Agustinho, James R. Vinyard, Takoha Harden, Sarah L. Bennett, Jose A. Arce-Cordero, Efstathios Sarmikasoglou, Anay D. Ravelo, Aneesa Bahman, Sarong So, Elis R. Vieira, Antonio P. Faciola

**Affiliations:** 1 Department of Population Health and Reproduction, School of Veterinary Medicine, University of California, Davis, CA 95616 USA; 2 Department of Animal, Veterinary, and Food Sciences, University of Idaho, Moscow, ID 83844 USA; 3 Department of Animal Sciences, University of Florida, Gainesville, FL 32611 USA; 4 Department of Agricultural and Environmental Sciences, Tuskegee University, Tuskegee, AL 36088 USA; 5 Department of Animal Science, Penn State University, University Park, PA 16803 USA; 6 Veterinary Population Medicine Department, University of Minnesota, St. Paul, MN 55108 USA; 7 Department of Animal Science, Khon Kaen University, Khon Kaen, Thailand; 8 Department of Animal Sciences, Tocantins Federal University, Palmas, Brazil; 9 Department of Animal Science, National University of Battambang, Battambang, Cambodia

**Keywords:** Microbial ecology, Symbiosis, Symbiosis

## Abstract

This study aimed to evaluate the effects of *Saccharomyces cerevisiae* and *Megasphaera elsdenii* as direct fed microbials (DFM) in beef cattle finishing diets to alleviate acute ruminal lactic acidosis in vitro. A dual-flow continuous culture system was used. Treatments were a Control, no DFM; YM1, *S. cerevisiae* and *M. elsdenii* strain 1; YM2, *S. cerevisiae* and *M. elsdenii* strain 2; and YMM, *S. cerevisiae* and half of the doses of *M. elsdenii* strain 1 and strain 2. Each DFM dose had a concentration of 1 × 10^8^ CFU/mL. Four experimental periods lasted 11 days each. For the non-acidotic days (day 1–8), diet contained 50:50 forage to concentrate ratio. For the challenge days (day 9–11), diet contained 10:90 forage to concentrate ratio. Acute ruminal acidosis was successfully established. No differences in pH, d-, l-, or total lactate were observed among treatments. Propionic acid increased in treatments containing DFM. For N metabolism, the YMM treatment decreased protein degradation and microbial protein synthesis. No treatment effects were observed on NH_3_–N concentration; however, efficiency of N utilization by ruminal bacteria was greater than 80% during the challenge period and NH_3_–N concentration was reduced to approximately 2 mg/dL as the challenge progressed.

## Introduction

Acute ruminal acidosis is a major digestive disorder in ruminants and remains one of the biggest challenges faced by the beef cattle industry^1–3^. The abrupt increase in consumption of rapidly fermentable carbohydrates along with a decrease in fibrous carbohydrates consumption (e.g., upon feedlot entrance and sorting behavior) causes an unbalance in ruminal fermentation^1,4^. In the rumen, rapidly fermentable carbohydrates are converted to volatile fatty acids (VFA) and lactic acid. Metabolism, absorption, and outflow of these acids may not be greater than their production^3^. Thus, accumulation of acids, especially lactic acid, may reduce ruminal pH below 5.2, which may impair ruminal fermentation^2^.

Lactic acid is a stronger acid compared to other VFA found in the rumen (pKa of 3.9 vs. 4.9, respectively) and ruminal bacteria that metabolize lactic acid (e.g., *Megasphaera elsdenii*) do not grow as fast as those that produce lactic acid (e.g., *Streptococcus bovis*; Nocek, 1997; Russell and Rychlik, 2001). Thus, one option to overcome acute ruminal acidosis is to decrease undissociated lactic acid in the rumen by supplementing microbial feed additives that can metabolize lactic acid. *Saccharomyces cerevisiae* has been a major direct fed microbial (DFM) studied to decrease undissociated lactic acid accumulation in the rumen^5^. In a meta-analysis of studies using *S. cerevisiae* as DFM to modulate ruminal fermentation, Desnoyers et al.^6^ reported that *S. cerevisiae* decreased ruminal lactate concentration and increased dry matter intake (DMI), ruminal pH, OM digestibility, and VFA in different species of ruminants. Because *S. cerevisiae* promotes a more reduced environment in the rumen through oxygen scavenging, acute ruminal acidosis may be alleviated with *S. cerevisiae* supplementation due to the proliferation of fibrolytic bacteria and fiber fermentation in the rumen^5^. Although *S. cerevisiae* contribute to lactic acid metabolization, the concentration of undissociated lactic acid during acute ruminal acidosis may require more than one DFM to effectively improve the ruminal environment.

Possible combinations may involve the use of lactic acid utilizing bacteria already found in the rumen, such as *M. elsdenii*^7^, *Selenomonas ruminantium*^8^, and *Propionibacterium freudenreichii*^9^. Specifically, *M. elsdenii* is the most promising since this bacterium is already the major lactic acid utilizing bacteria in the rumen, accounting for up to 80% of all lactic acid fermentation to propionic acid under normal conditions^10^. *Megasphaera elsdenii* has been reported to ferment lactic acid until the latter becomes depleted, as lactic acid is fermented approximately 6 times faster than glucose by *M. elsdenii*^11,12^; thus, making this bacterium a strong DFM candidate to be used during acute ruminal acidosis. Despite some strains of *M. elsdenii* being patented as DFM to prevent acidosis^13,14^, few other strains have successfully ameliorated acute ruminal acidosis conditions^15,16^.

Therefore, the objectives of this study were to: (1) induce acute ruminal lactic acidosis in a dual-flow continuous culture system; and (2) to evaluate the effects of feeding *S. cerevisiae* in combination with two newly isolated strains of *M. elsdenii* (isolated from the rumen of beef cattle under acute acidosis) as DFM during acute ruminal lactic acidosis conditions. The hypothesis was that an acute ruminal acidosis scenario could successfully be induced in vitro by abruptly changing the diets fed to dual-flow continuous culture fermenters. The second hypothesis was that a combination of *S. cerevisiae* with both *M. elsdenii* strains would considerably decrease the lactic acid accumulation during this change of diets (challenge) and improve ruminal fermentation. Herein, we present a model for simulating ruminal acidosis and describe methods to evaluate the possible effects of DFM to ameliorate such challenging conditions.

## Results and discussion

**Figure 1:**
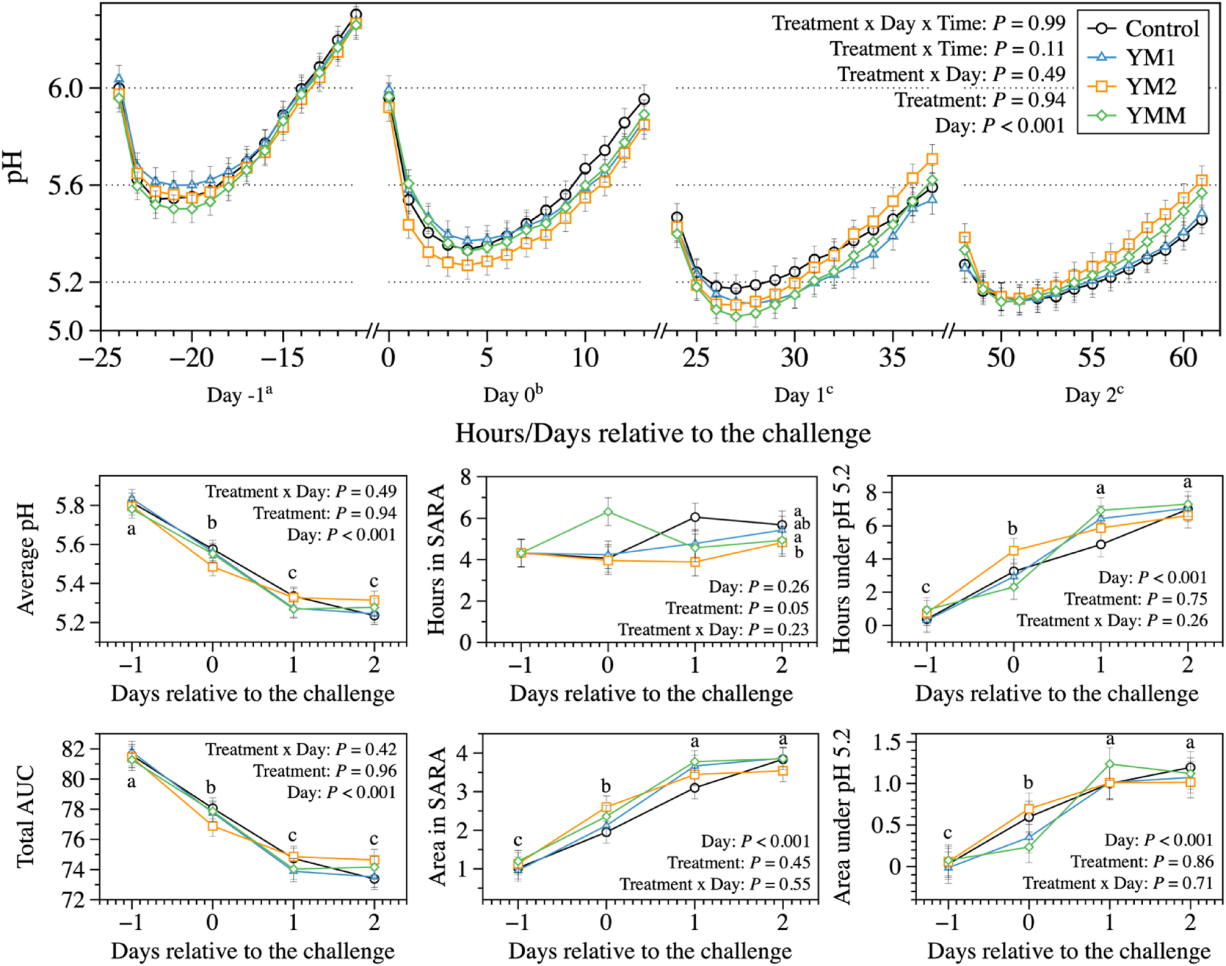
Dynamics of fermentation pH after the morning feeding, area under the curve (AUC), and time in SARA and below pH 5.2 on days 8, 9, 10, and 11 of each period to represents the days − 1, 0, 1, and 2 relative to the challenge; day 7 was used as a covariate to the model. All treatments had the same basal diet (1 mL of each DFM was applied per day at 1 × 10^8^ cfu/mL); treatments were: Control, carrier of additives without DFM; YM1, *S. cerevisiae* and *M. elsdenii* strain 1; YM1, *S. cerevisiae* and *M. elsdenii* strain 2; YMM, *S. cerevisiae* and *M. elsdenii* strain 1 (1/2 dose) and strain 2 (1/2 dose). Statistical differences were declared at *P* ≤ 0.05 or as a trend to be different if *P* > 0.05 and < 0.10.

A recently published meta-analysis with 155 published articles has demonstrated the soundness of the dual-flow continuous culture system when compared to in vivo conditions^17^. Thus, one of the goals of the study was to create a scenario of acute ruminal acidosis in the dual-flow continuous culture fermenters. Despite the soundness of this methodology, findings should be carefully considered before in vivo applications are proposed. On the other hand, because this system allows a precise control of feed intake and ruminal dilution rates (both liquid and solid flows) that are not feasible in vivo, it allow us to isolate such effects to better evaluate this nutritional disorder. Although major reviews in the literature differ in what is considered an acceptable pH threshold for acute ruminal acidosis (pH < 5.2 in Owens et al.^1^ vs. pH < 5.0 in Nagaraja and Titgemeyer^2^), there is a consensus that acute ruminal acidosis is characterized not only by the occurrence but also the extent to which pH is below normal fermentation conditions (average daily pH < 5.8)^2,18,19^. In Fig. 1, the pH is shown to have reached subacute ruminal acidosis (SARA) conditions on the day diets were changed and reached the threshold of 5.2 during days 1 and 2 of the challenge period. The average pH, hours under pH 5.2, and area under pH 5.2 dropped below normal fermentation conditions (pH < 5.6) throughout the challenge period, staying in acidosis conditions in days 1 and 2 relative to the challenge. These are important indicators in the dual-flow continuous culture system in comparison to in vivo models because they represent an extended exposure to the problem, which in vivo would have the potential to promote considerable physiological responses^1,2,20^. Overall, there was no significant interaction or treatment effect on improving the acute ruminal acidosis conditions, despite YMM treatment being the only treatment to numerically overcome the thresholds of acute and subacute ruminal acidosis pH by the end of days 1 and 2 relative to the challenge. Given that *M. elsdenii* metabolism has been reported to be more regulated by pH than substrate concentration^21^, there is a possibility that the mix YMM slightly downregulated this mechanism and worked better in such conditions.

Given the lack of effects on ameliorating the acidosis challenge, the *S. cerevisiae* and new strains of *M. elsdenii* used in the study were probably not efficient at reducing the lactate concentration during fermentation. The strains of *M. elsdenii* used in the present study were isolated from the rumen during acute ruminal acidosis; thus, these strains were expected to have ameliorated such conditions. The reason that *M. elsdenii* was not efficient in decreasing lactate concentration may be due to the difficulty of *M. elsdenii* population to establish itself in the ruminal fluid, as it has been reported in vivo^22^. Past studies reported that *M. elsdenii* may not establish well in the rumen even if they were isolated from the rumen, possibly because of host specificity^23^. Because the current study was performed in vitro, the microbiome during the pre-challenge period may have established a dynamic that was resistant to change. Furthermore, the competition for substrates with the native microbiota may decrease the likelihood of *M. elsdenii* and *S. cerevisiae* survival, as they compete for substrates with other bacterial groups^24^. The original microbial population may have prevented the proliferation of other populations such as the tested DFM. Another factor for the lack of establishment of the *M. elsdenii* population may have been the frequency of dosing the fermenters. In Weimer et al.^22^, different dosing times were tested, and even when *M. elsdenii* strains were dosed 4 times in 5 days, *M. elsdenii* strains returned to their original low ruminal concentration rapidly after infusion. Trying to avoid this problem, in our study the DFM were dosed in the study twice a day, which still may not have been ideal. Lastly, it is important to recognize that other microorganisms may also metabolize ruminal lactic acid in rumen and the contribution of *M. elsdenii* and *S. cerevisiae* to its metabolism may have been overestimated during acute ruminal acidosis conditions^2,3,21^.

There was a consistent effect of day in the study; most of the acute acidotic conditions were established just one day after the beginning of the challenge [days 1 and 2 relative to the challenge (Fig. 1)]. The establishment of acidotic conditions one day after the change of diets may have happened due to a few reasons: first, one of the advantages of the dual-flow continuous culture system is that it precisely regulates the fluid and particulate flow rates out of the fermenter (e.g., rumen model). We selected this flow rates based on reported finishing beef cattle ruminal dilution rates. Although the system precisely regulates digesta flow rates to mimic in vivo conditions based of specific animal models, such precise regulation also minimize individual animal effects that could undergo such challenges more rapidly or slower. The advantage is that by controlling feed intake and digesta flow rates, we are able to isolate the ruminal function, which was our main goal and cannot be done in vivo. Thus, as in an in vivo model, the response to the treatments may be variable due to changes in feed intake, digesta flow rates, environmental and other factors, here we were able to isolate and uniquely test these treatments in an acute ruminal acidosis scenario. The second reason is regarding the dilution effect from the presence of NDF residues in the fermentation remnants from the non-acidotic diet (Table 1). Although dietary NDF is associated with greater chewing activity and buffering capacity of the rumen^19,25,26^, the system used in this study had a continuous infusion of artificial saliva throughout the experiment. Therefore, these conditions may have contributed to the presence of a mat still observed in the first day of the challenge. This mat works as a micro environment with elevated pH that can optimize the degradation of structural carbohydrates^25^. The mat was visible on day 0 but absent on days 1 and 2 relative to the challenge (see Supplementary Figs. 1 and 2).

**Table 1:** Effects of direct-fed microbials (DFM) on true nutrient and microbial outflow in dual-flow continuous culture system (DM basis).

Item, g/day	Treatments^A^				SEM	*P* values^B^		
	Control	YM1	YM2	YMM		Treatment	Day	Treatment × day
**True dietary outflow**								
DM	40.5	40.2	41.4	42.3	2.68	0.68	< 0.01	0.80
OM	33.7	33.6	33.4	35.3	2.31	0.25	< 0.01	0.90
CP	5.85^b^	6.07^a,b^	5.95^b^	7.24^a^	0.72	0.02	< 0.01	0.46
NDF	10.4	10.8	10.6	10.8	0.95	0.76	< 0.01	0.82
ADF	3.81	4.18	4.19	4.23	0.22	0.49	< 0.01	0.82
Starch	5.29	4.91	4.66	5.04	0.52	0.84	0.61	0.85
**Microbial outflow**								
Microbial DM	16.3	18.0	15.6	15.4	0.96	0.12	< 0.01	0.93
Microbial OM	13.6^a,b^	15.2^a^	13.1^a,b^	12.7^b^	0.81	0.10	< 0.01	0.93
Microbial CP	8.31^a,b^	8.94^a^	8.06^a,b^	7.70^b^	0.41	0.09	< 0.01	0.90
Microbial glycogen, mg/day	1047	1154	902	747	119	0.11	0.01	0.64

^A^All treatments had the same basal diet (1 mL of each DFM was applied per day at 1 × 10^8^ cfu/mL); treatments were: Control, carrier of additives without DFM; YM1, *S. cerevisiae* and *M. elsdenii* strain 1; YM1, *S. cerevisiae* and *M. elsdenii* strain 2; YMM, *S. cerevisiae* and *M. elsdenii* strain 1 (1/2 dose) and strain 2 (1/2 dose).

^B^Statistical differences were declared at *P* ≤ 0.05 or as a trend to be different if *P* > 0.05 and < 0.10.

**Figure 2:**
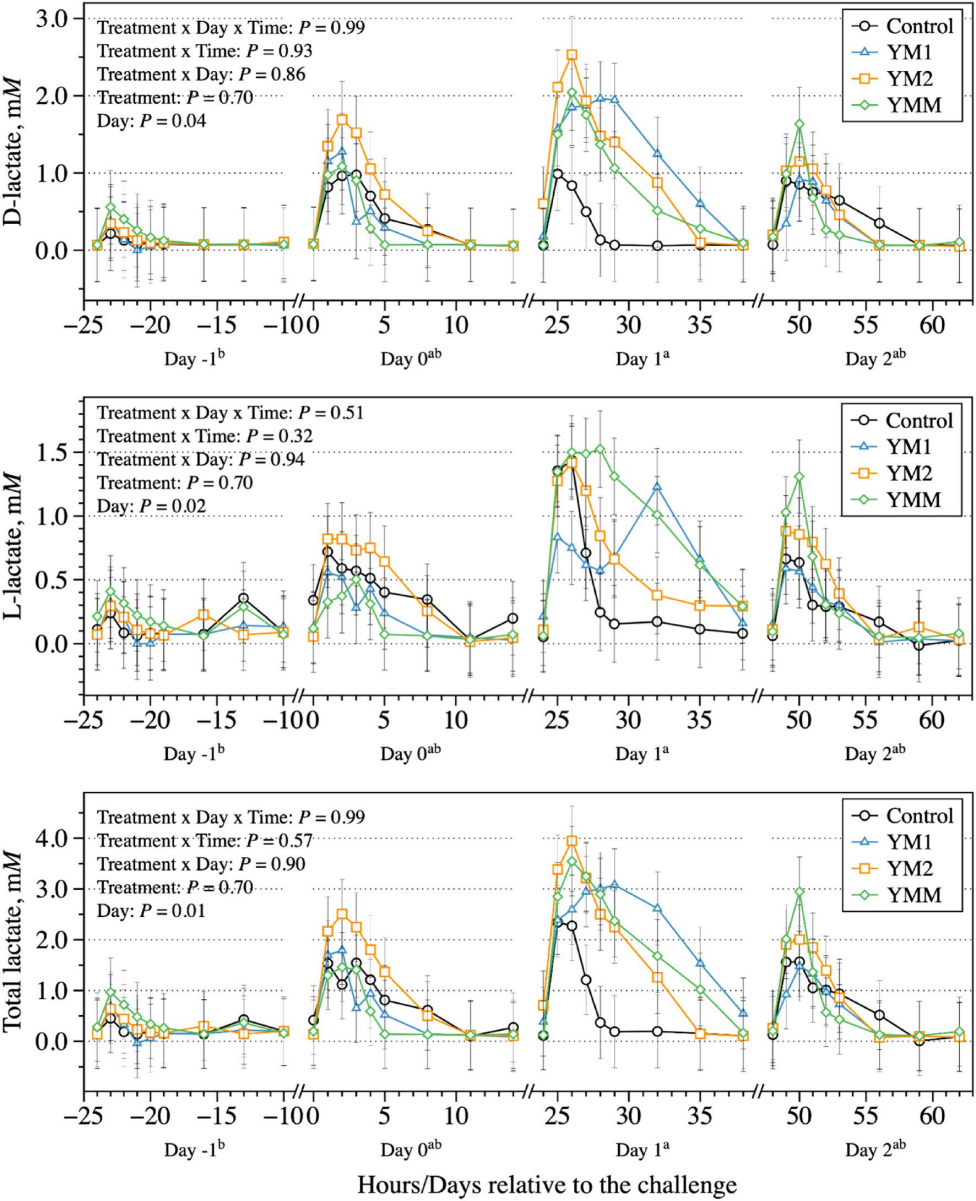
Dynamics of d-lactate, l-lactate, and total lactate concentration after the morning feeding on days 8, 9, 10, and 11 of each period to represents the days − 1, 0, 1, and 2 relative to the challenge; day 7 was used as a covariate to the model. All treatments had the same basal diet (1 mL of each DFM was applied per day at 1 × 10^8^ cfu/mL); treatments were: Control, carrier of additives without DFM; YM1, *S. cerevisiae* and *M. elsdenii* strain 1; YM1, *S. cerevisiae* and *M. elsdenii* strain 2; YMM, *S. cerevisiae* and *M. elsdenii* strain 1 (1/2 dose) and strain 2 (1/2 dose). Statistical differences were declared at *P* ≤ 0.05 or as a trend to be different if *P* > 0.05 and < 0.10.

**Figure 3:**
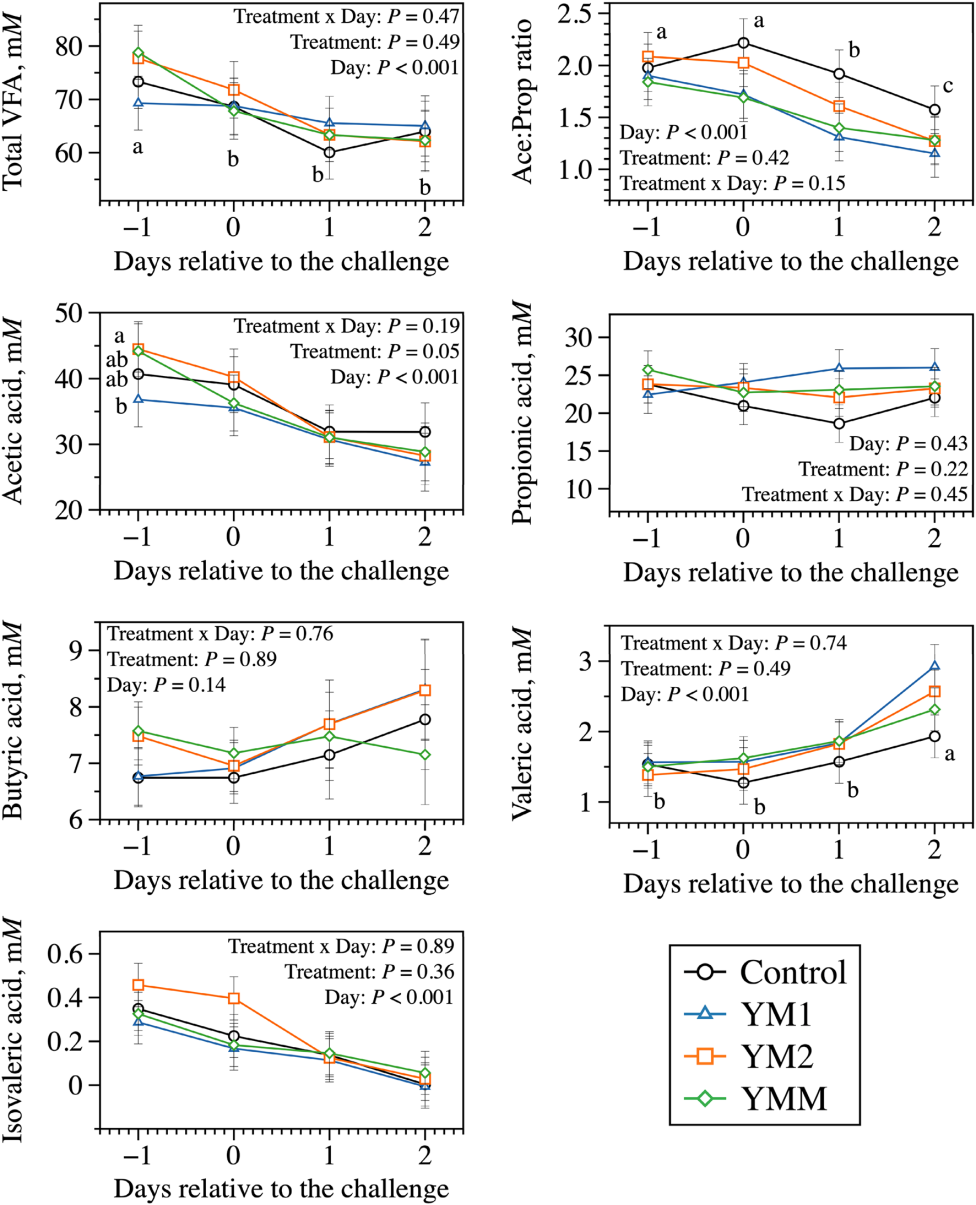
Dynamics of volatile fatty acids (VFA) concentration after the morning feeding on days 8, 9, 10, and 11 of each period to represents the days − 1, 0, 1, and 2 relative to the challenge; day 7 was used as a covariate to the model. All treatments had the same basal diet (1 mL of each DFM was applied per day at 1 × 10^8^ cfu/mL); treatments were: Control, carrier of additives without DFM; YM1, *S. cerevisiae* and *M. elsdenii* strain 1; YM1, *S. cerevisiae* and *M. elsdenii* strain 2; YMM, *S. cerevisiae* and *M. elsdenii* strain 1 (1/2 dose) and strain 2 (1/2 dose). Statistical differences were declared at *P* ≤ 0.05 or as a trend to be different if *P* > 0.05 and < 0.10.

Another possible explanation for the establishment of acidotic conditions a day after the change of diets is regarding to when the peak of total lactate concentration happened, which despite the small difference between challenge days, it was observed mostly on day 1 relative to the challenge but not day 0 (Fig. 2). Based on previous literature, it is expected that during a ruminal acidosis challenge bacteria that ferment non-structural carbohydrates to lactic acid (e.g., lactic acid producing bacteria) will quickly reproduce given favorable conditions^27,28^. On the other hand, bacteria that ferment lactate to other VFA such as propionic acid (e.g., lactic acid utilizing bacteria) are expected to have a longer doubling time in the rumen^27,29^; this lack of synchronization between these bacterial groups results in the accumulation of lactate (stronger acid when undissociated) and, consequently, a pH drop^2^. There is the possibility that bacteria need to adjust to the increased substrate to upregulate their metabolism and produce a measurable shift, which could explain the delay in establishing such conditions. Furthermore, the current results suggest that despite a possible increase in lactic acid producing bacteria during fermentation, the total VFA concentration (Fig. 3) was depressed across the challenge days which represents a greater lack of synchrony between bacterial groups as the challenge progressed.

Interestingly, because the dual-flow continuous culture system removes end-products of fermentation at the same rate, the concentration of end-products of fermentation is expected to be similar to the production of these end-products. Therefore, this system allows to better understand the production of lactic acid isomers during acidotic conditions, which has been proposed to be greater for l-lactic acid compared to d-lactic acid^30^. During the peak of lactic acid production (day 1 relative to the challenge), the control treatment had greater l-lactate concentration than d-lactate concentration (Fig. 2). However, the treatments containing DFM had the opposite pattern, and d-lactate concentration was greater than l-lactate concentration. Although no interaction or treatment effect for lactate concentration was observed, data suggests the treatments containing DFM had greater and more prolonged production of lactic acid compared to the control, which was mostly due to the greater d-lactic acid production. According to Harmon et al.^31^, l-lactic acid may have a higher absorption rate in the rumen compared to d-lactic acid, which suggests that if their proportions are inverted during fermentation the latter may accumulate and negatively affect pH in an in vivo setting. It is important to note; however, that despite lactic acid being an important component affecting ruminal pH, especially during ruminal acidosis conditions^2,21^, and a stronger acid compared to other ruminal VFA (pKa of lactic acid = 3.9 vs. ruminal VFA = 4.9), we expected a greater concentration of this metabolite in our study. Thus, the overall low concentration of lactic acid we found in this system that isolates other factors such as feed intake, digesta flow rates, saliva volume, variation in urea recycling rate, and others, suggests such contribution of lactic acid to acute ruminal acidosis could have been overestimated in previous in vivo studies and warrants further research.

Despite the lack of treatment effects on lactic acid concentration, the final acetic acid concentration was greater for the control compared to the DFM treatments, while the opposite happened to propionic acid concentration (Table 2). The ratio of acetic to propionic acid was also affected by the DFM inclusion, which the control had the greatest ratio compared to the other treatments. In fact, the treatments of the current study had the goal of metabolizing lactate to VFA, especially propionic acid^10,32^. However, we believe these treatments had only a mild effect on reducing lactic acid concentration and producing propionic acid, as no effects of treatment were observed in lactate concentrations (Fig. 2) and minor effects were observed on acetic and propionic acid concentrations during snapshot collections during the challenge (Fig. 3).

**Table 2:** Effects of direct-fed microbials (DFM) on volatile fatty acids (VFA) concentration on a 24 h pool from feeding in a dual-flow continuous culture system.

Item	Treatments^A^				SEM	*P* values^B^		
	Control	YM1	YM2	YMM		Treatment	Day	Treatment × day
Total VFA, mM	58.8	54.2	60.3	55.2	4.42	0.75	0.72	0.33
**VFA, % of total VFA**								
Acetic acid	55.2^a^	47.6^b^	52.5^a,b^	50.9^a,b^	3.15	0.01	< 0.01	0.32
Propionic acid	31.8^b^	38.8^a^	34.6^a,b^	36.1^a,b^	2.84	0.01	< 0.01	0.38
Butyric acid	10.7	10.5	10.4	10.4	0.57	0.96	< 0.01	0.77
Valeric acid	2.13	2.41	2.45	2.32	0.30	0.86	< 0.01	0.40
*Iso-*valeric acid	0.18	0.17	0.15	0.17	0.10	0.99	< 0.01	0.85
Acetic:propionic acid	1.97^a^	1.38^b^	1.73^a,b^	1.55^a,b^	0.22	0.02	< 0.01	0.06

^A^All treatments had the same basal diet (1 mL of each DFM was applied per day at 1 × 10^8^ cfu/mL); treatments were: Control, carrier of additives without DFM; YM1, *S. cerevisiae* and *M. elsdenii* strain 1; YM1, *S. cerevisiae* and *M. elsdenii* strain 2; YMM, *S. cerevisiae* and *M. elsdenii* strain 1 (1/2 dose) and strain 2 (1/2 dose).

^B^Statistical differences were declared at *P* ≤ 0.05 or as a trend to be different if *P* > 0.05 and < 0.10.

**Figure 4:**
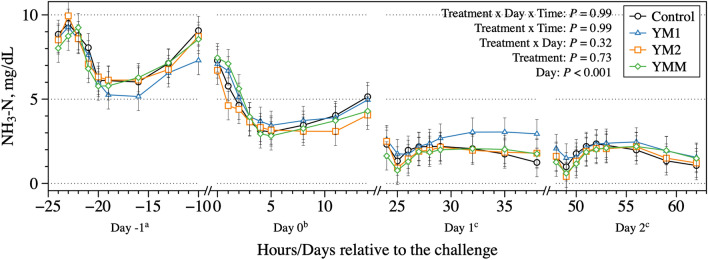
Dynamics of NH_3_–N concentration after the morning feeding on days 8, 9, 10, and 11 of each period to represents the days − 1, 0, 1, and 2 relative to the challenge; day 7 was used as a covariate to the model. All treatments had the same basal diet (1 mL of each DFM was applied per day at 1 × 10^8^ cfu/mL); treatments were: Control, carrier of additives without DFM; YM1, *S. cerevisiae* and *M. elsdenii* strain 1; YM1, *S. cerevisiae* and *M. elsdenii* strain 2; YMM, *S. cerevisiae* and *M. elsdenii* strain 1 (1/2 dose) and strain 2 (1/2 dose). Statistical differences were declared at *P* ≤ 0.05 or as a trend to be different if *P* > 0.05 and < 0.10.

Another unique feature to study in dual-flow continuous culture fermenters is the possibility of isolating the effect of urea recycling from ruminal NH_3_–N concentration. Urea recycling was simulated in this system through a constant rate of urea infusion with the artificial saliva; thus, changes in NH_3_–N concentration during fermentation were not due to changes in the rate of urea recycling. Herein, a shift in NH_3_–N concentration was observed when diets were switched, from 5 to 10 mg/dL with the non-acidotic diet, to 3 to 8 mg/dL on day 0, and later ranging from 1 to 3 mg/dL after acidotic conditions were established (days 1 and 2; Fig. 4). According to Satter and Slyter^33^, a minimum of 2 mg of NH_3_–N/dL is necessary in order for microbial fermentation to proceed. Ammonia–N concentration levels below normal fermentation conditions may reduce protein synthesis and growth of some microorganisms (e.g., fibrolytic bacteria)^33^. Although the efficiency of N utilization by ruminal microorganisms was high in the study (> 80%; Table 3), some microorganisms such as lactic acid-producing bacteria (LAB) that thrive in low pH conditions^2^ may have increased efficiency in utilizing NH_3_–N for protein synthesis. Because microorganisms that may have their growth impaired in such conditions produce weaker VFA (e.g., acetic and butyric acid) compared to lactic acid from LAB bacteria, an increase in the rate of urea recycling would be fundamental to keep the growth of the former microbial populations. In an in vivo setting, the low NH_3_–N concentration in the rumen would theoretically be compensated by an increase in urea recycling through the saliva and blood^34,35^. These findings suggests that some animal variation in triggering the quick increase in urea recycling may represent a higher risk of acute ruminal acidosis; a condition that may worth further evaluation.

**Table 3:** Effects of direct-fed microbials (DFM) on ruminal N metabolism in a dual-flow continuous culture system (DM basis).

Item, g/day otherwise stated	Treatments^A^				SEM	*P* values^B^		
	Control	YM1	YM2	YMM		Treatment	Day	Treatment × day
**N flows**								
Total N	2.44	2.50	2.43	2.53	0.10	0.39	< 0.01	0.63
NH_3_-N	0.14	0.14	0.15	0.14	0.01	0.36	< 0.01	0.12
NAN^C^	2.30	2.36	2.28	2.39	0.11	0.23	< 0.01	0.48
Microbial-N	1.33^a,b^	1.43^a^	1.29^a,b^	1.23^b^	0.07	0.09	< 0.01	0.90
Dietary-N	0.94^a^	0.97^a^	0.95^a^	1.16^b^	0.12	0.01	< 0.01	0.48
Dietary N, % of diet CP	36.1^a^	37.5^a^	36.7^a^	44.7^b^	4.45	0.01	< 0.01	0.48
**Efficiency**								
Efficiency of N use^D^, %	83.9	87.8	82.1	88.1	5.45	0.55	0.05	0.99
Microbial efficiency^E^, %	19.9^a,b^	21.4^a^	19.4^a,b^	18.9^b^	0.92	0.09	0.42	0.95

^A^All treatments had the same basal diet (1 mL of each DFM was applied per day at 1 × 10^8^ cfu/mL); treatments were: Control, carrier of additives without DFM; YM1, *S. cerevisiae* and *M. elsdenii* strain 1; YM1, *S. cerevisiae* and *M. elsdenii* strain 2; YMM, *S. cerevisiae* and *M. elsdenii* strain 1 (1/2 dose) and strain 2 (1/2 dose).

^B^Statistical differences were declared at *P* ≤ 0.05 or as a trend to be different if *P* > 0.05 and < 0.10.

^C^NAN = Non ammonia nitrogen.

^D^Efficiency of N use = g of microbial N/g of available N (Bach and Stern, 1999).

^E^Bacterial efficiency = g of microbial N/kg of OM truly digested.

Similarly, the low N availability during the challenge period may have changed the pattern of substrate utilization by *M. elsdenii*^12,21,22^. Although *M. elsdenii* ferments lactic acid faster than glucose, this bacterium has been reported to have a greater growth yield on glucose than lactic acid^12^. Combined with the low N availability, a change in substrate fermentation pattern may have also happened in the current study, decreasing the efficacy of *M. elsdenii* on ameliorating acidosis and reducing the undissociated lactic acid load during fermentation.

The outflow of nutrients from fermentation was also measured during the challenge to understand if DFM would improve nutrient digestibility. Nutrient outflow is reported in Table 1; no interaction or treatment effect were observed. Except for the starch outflow, all other nutrients had a day effect (data not shown) possibly because of residue of the non-acidotic diet present in the first hours of the challenge day. The lack of a day effect for the starch flow was expected, as non-fibrous carbohydrates are expected to be quickly degraded in the rumen^1,2^. For DM, OM, and CP, day 0 had the greatest outflow, while for NDF and ADF, the outflow continuously decreased during the challenge days. The outflow of microbial DM, OM, CP, and glycogen were higher for the days 1 and 2 compared to day 0 possibly because of a higher efficiency of N utilization as more readily fermentable carbohydrates were available. There was a pattern of decreasing CP digestibility when the YMM treatment was used, as seen by the greater true CP and lower microbial OM and CP outflows from fermentation (Table 1) as well as a decrease in microbial efficiency (Table 3). The literature is scarce about the effect of lactic acid utilizing bacteria on ruminal N metabolism. However, as these new *M. elsdenii* strains had not yet been further evaluated due to recent extraction and culture from an acidotic rumen, a possible explanation is the competition of these strains towards substrates utilized by other bacterial groups^24^. These new strains may have competed for protein with other bacterial groups; possibly explaining the reduced protein degradation for this treatment. Furthermore, having a treatment without yeast (or only yeast) presents a clearer picture on the specific effects of *M. elsdenii* alone or even how they complement each other; however, given the limitations of the current study, this was not evaluated, and further research is warranted.

In conclusion, this study was successful at inducing acute ruminal acidosis in vitro, as shown by the decrease of fermentation pH below 5.2 for an extended period in dual-flow continuous culture fermenters. Furthermore, by isolating factors that cannot be isolated in vivo due to individual animal variation, we were able to evaluate the onset of acute ruminal acidosis more clearly and propose potential gaps in knowledge, which are less likely to be observed in vivo. The DFM treatments had a different pattern of lactic acid production compared to the control; more d-lactic acid was produced than l-lactic acid during fermentation. Treatments promoted a mild increase in propionic acid during the challenge, but no reduction in lactate concentrations were observed. Because the lactic acid concentration was less than we expected in the current study, the data indicates that other factors than lactic acid may be of more importance in the onset of acute ruminal acidosis than previously reported. This in vitro system allowed to observe a reduced NH_3_–N concentration, which was near deficiency; a situation that may not always be observed in vivo due to urea recycling stimulation and has a great likelihood to be part of the onset of acute ruminal acidosis. Lastly, despite the lack of major effects from the DFM tested in our study, the mix of YMM containing all microorganisms used as DFM reduced protein degradation while being the only treatment overcoming the challenge by the last day of fermentation. The findings reported in this study regarding the importance of N on acute ruminal acidosis highlights once again other important factors besides lactic acid that may needs to be considered in future research aiming to ameliorate such relevant nutritional disorder in the beef and dairy cattle industry.

## Materials and methods

All experimental procedures involving the animals used as donors of rumen fluid in the study were conducted under protocols approved by the University of Florida Institutional Animal Care and Use Committee (IACUC #202009849). Moreover, all methods were performed in accordance with the IACUC guidelines and regulations. The following study is reported in accordance with ARRIVE guidelines.

### Experimental design and treatments

Eight dual-flow continuous culture fermenters (1820 mL) similar to those developed by Hoover et al.^36^ and modified by Del Bianco Benedeti et al.^37^, Silva et al.^38^, and Paula et al.^39^ were used in the study to simulate ruminal fermentation. Each of the fermenters were considered an experimental unit and they were arranged in a replicated 4 × 4 Latin square design. There were 4 experimental periods, each consisting of 11 days of fermentation. Acute ruminal acidosis conditions were created in the fermenters by feeding them from days 1–8 a diet that would not cause acute ruminal acidosis (non-acidotic diet), and from days 9–11 by feeding the fermenters a high-grain diet (challenge diet) to promote acidotic conditions. Both diets were formulated similarly to those of finishing beef cattle steers^19^ and are presented in Table 4.

**Table 4:** Ingredient and chemical composition of the experimental diets (% of DM unless otherwise stated).

Item	Non-acidotic diet	Challenge diet
	(days 1–8)	(days 9–11)
Alfalfa hay	50.0	10.0
Ground corn	45.1	78.4
Solvent soybean meal 48% CP	1.90	8.60
Vitamin and mineral premix	3.00	3.00
**Chemical composition**		
Forage:concentrate	50/50	90/10
OM	89.6	94.0
CP	14.7	13.5
NDF	25.4	12.0
ADF	17.2	5.67
NFC^a^	46.7	65.4
Starch	33.8	57.8
Ether extract	2.70	3.29
TDN^b^	71.0	80.6

Ingredients were milled to pass through a 1-mm screen for chemical analysis and through a 2-mm screen for experiments.

^a^NFC (non-fiber carbohydrates) = 100 − (% NDF + % CP + % ether extract + % ash).

^b^TDN (total digestible nutrients) = dCP + (dEther extract × 2.25) + dNDF + dNSC, which NSC (non-structural carbohydrates) = 100 – CP – EE – NDF − ash.

One day before switching the diets (day 8), the experimental treatments (direct fed microbials) were infused in the fermenters during the morning feeding time and every feeding time after that until day 11. The treatments were a Control, containing only the carrier used in all treatments (aqueous solution containing 15% glycerol); YM1, the carrier plus *S. cerevisiae* and *M. elsdenii strain 1*; YM2, the carrier plus *S. cerevisiae* and *M. elsdenii strain 2*; and YMM, the carrier plus *S. cerevisiae* and half of the doses of *M. elsdenii strain 1* and *strain 2*. Each DFM dose had a concentration of 1 × 10^8^ CFU/mL. All treatments and their composition are presented in Table 5.

**Table 5:** Direct-fed microbials (DFM) composition and inclusion rate in the dual-flow continuous culture system.

Item	Daily inclusion rate (mL)	DFM composition	CFU/mL
Control	2.000	Only carrier without DFM	–
YM1	1.000	*Saccharomyces cerevisiae*	1 × 10^8^
	1.000	*Megasphaera elsdenii* strain 1	1 × 10^8^
YM2	1.000	*S. cerevisiae*	1 × 10^8^
	1.000	*M. elsdenii* strain 2	1 × 10^8^
YMM	1.000	*S. cerevisiae*	1 × 10^8^
	0.500	*M. elsdenii* strain 1	1 × 10^8^
	0.500	*M. elsdenii* strain 2	1 × 10^8^

Dietary ingredients used throughout the entire experiment were acquired from the same batch of feed and later ground in the same day. Ingredients were ground to pass a 2-mm sieve using a Wiley Mill (Arthur H. Thomas Co., Philadelphia, PA), and a subsample of 500 g from each of the ingredients was ground to pass a 1-mm sieve for chemical analyses. All dietary ingredients were properly stored in a temperature- and humidity-controlled environment. For every feeding time, diets were individually prepared for each fermenter by weighing the dietary ingredient separately and storing them in sealed plastic bags (14 × 8 cm).

### Dual-flow continuous culture system

Three Black Angus steers averaging 630 kg of BW and fitted with permanent, 10 cm-ruminal cannulas (Bar Diamond, Inc., Parma, ID), were used as donors of ruminal content. Animals were kept from 2 weeks prior to the first collection until the end of the study in the same non-acidotic diet fed to fermenters. On the inoculation day, ruminal content was collected from all 3 cannulated steers at 2 h after the morning feeding from the anterior, posterior, caudal, and ventral areas of the rumen. Ruminal content was strained through 4 layers of cheesecloth into pre-warmed insulated vessels and brought to the lab (~ 5 min away). At the lab, ruminal content from all animals were equally homogenized before being inoculated into the fermenters. Ruminal content was then poured into the pre-warmed fermenters up to effluent limit. Fermentation conditions were maintained by setting the fermenters to an agitation of 100 rpm, temperature of 39 °C, and an infusion of N_2_ in the fermentation content and in the headspace of the fermenters of 200 mL of N_2_/min.

Independently of the diet, the fermenters were fed 107 g of DM per day equally divided into two meals (7:00 and 21:00 h). Artificial saliva was prepared according to Weller and Pilgrim^40^ and was continuously infused in the fermenters as the buffer for ruminal fermentation. To simulate the urea recycling in the rumen, urea was added to the artificial saliva at a rate of 0.4 g/L. Because the dual-flow continuous culture system allows the passage rates of ruminal content out of fermenters to be pre-determined, the dilution rate was set at a rate of 10%/h while the passage rate of solids was set at a rate of 5%/h; all based on the fermenter’s volume and similar to those observed in finishing beef steers. These rates were adjusted by two mechanisms: (1) the continuous infusion of artificial saliva through a peristaltic pump (liquid portion) along with the diet (solid portion) into the fermenters; and (2) the continuous output of fermentation content through two ports, being the first the removal of filtered fermentation liquid (500 µm wire mesh) from the fermenters at a rate of 5%/h of the fermenter’s volume by another peristaltic pump, and the second being the continuous outflow by gravity of fermentation content from the difference between mechanism 1 and the removal of filtered fermentation liquid from the fermenter. The content from both output ports of each fermenter were collected in two separated 4.3 L plastic containers.

### Experimental procedures and sample collection

Throughout the entire experiment, fermentation pH was manually measured every hour for 14 h to represent a full fermentation day, starting at right before the morning feeding time (0700 h) and stopping right before the night feeding time (2100 h). The feeding times were performed considering an interval of 14 h during the day (0700 until 2100 h) and 10 h during the night (2100 until 0700 h) for the lowest pH of a full day to take place 3–4 h after the morning feeding time (based on a pre-trial that we performed before the study). These pH measurements were performed with a portable pH meter (Thermo Scientific Orion Star A121) through a port in the headspace of the fermenters. Except for sampling days, the effluent content in the plastic containers were weighted and discarded daily right before the morning feeding time.

Right before the morning feeding time on day 5 of each period, the effluent containers from each fermenter were mixed, and a sample of 500 g was collected for ^15^N natural abundance analysis; this sample was named background. Then, the fermenters were enriched with ^15^N as a marker of microbial protein synthesis by first bringing the ^15^N concentration to a steady state in the fermenters and later by keeping the infusion of ^15^N constant in there^40^. Therefore, a pulse dose of 10.2% excess of (^15^NH_4_)_2_SO_4_ was infused into the fermenters before the morning feeding time on day 5, and urea in the artificial saliva was partially replaced with an isonitrogenous amount of (^15^NH_4_)_2_SO_4_ (Sigma-Aldrich Co., St. Louis, MO); this artificial saliva labeled with ^15^N was used from day 5 until the end of each experimental period.

On day 6 and throughout the collection period, the effluent containers were kept in a chilled water bath (< 4 °C) to stop microbial activity of the effluent contents whenever those left the fermenters. On day 7, under the non-acidotic diet and a day before the treatments were applied, the pH was measured every hour between the morning and night feeding times (14 h), and two samples (10 and 2 mL) were collected from inside the fermenters right before the morning feeding and at 1, 2, 3, 4, 5, 8, 11, and 14 h after feeding for NH_3_–N, VFA and lactate concentration analyses in the fermenters. The sample for NH_3_–N and VFA (10 mL) was collected by filtering the fermentation content in 4 layers of cheesecloth and immediately acidifying the liquid in 0.1% of a 50% H_2_SO_4_ solution. The sample for lactate (2 mL), although not acidified was also collected by filtering the fermentation content in 4 layers of cheesecloth; both samples were stored at – 20 °C for further analysis. These samples were used as the covariate for their respective variables later in the statistical analyses.

From days 8–11, the treatments were then applied during every feeding time. Treatments were kept in screw cap vials separated per feeding time and stored in a – 80 °C freezer until the time each vial was used. Fifteen minutes before each feeding time, the vials to be used for that specific time were thawed in warm water, and the culture of each vial was resuspended 5 times using a sterile pipette tip. Then, following the doses reported in Table 5, each treatment was applied to their respective fermenters along with the diet. For day 8, the same collections of pH, NH_3_–N, VFA, and lactate were performed, and the data were used as an overview of the fermentation pattern for the fermenters while in the non-acidotic diet (baseline).

Finally, from days 9–11, the non-acidotic diet was replaced by the challenge diet and fed to the fermenters while receiving their respective treatments. The same sampling schedule from days 7 and 8 for pH, NH_3_–N, VFA, and lactate was performed to evaluate both acute ruminal acidosis scenario and the fermentation response to the treatments. Also, with the goal of evaluating how the treatments could affect the ruminal N metabolism and true digestibility of nutrients after a complete day of fermentation, a sample of 500 g was collected from each fermenter’s effluent (mix of both containers) and stored at −20 °C for further analysis. Similarly, a sample of 10 mL was collected following the same sample preparation described earlier for NH_3_–N and VFA to further explore the final NH_3_–N daily outflow and the VFA concentration representing a day of fermentation. These collections were performed from the effluents before the following morning feeding time to account for a complete experimental day.

At the end of the last day of each experimental period, the entire content of each fermenter was used for bacterial isolation following the procedures described by Krizsan et al.^41^ and Brandão et al.^42^. Briefly, the content was blended with a 200 mL NaCl solution for 30 s, then filtered through 4 layers of cheesecloth, and the retained solids rinsed with an extra 200 mL of the NaCl solution. The filtered content was then centrifuged three times under 4 °C until a clean bacterial pellet was obtained. The pellet was stored at − 20 °C for further analysis as well.

### Chemical analyses

The samples for NH_3_-N and VFA analyses were centrifuged at 10,000×*g* for 15 min at 4 °C. A subsample of the supernatant was used for the determination of NH_3_–N. The NH_3_–N analysis was performed according to Broderick and Kang^43^ with an adaptation for plate readers^44^. The remaining supernatant was centrifuged again at 10,000×*g* for 15 min at 4 °C, being the newer supernatant filtered through a cellulose acetate syringe filter (SF14485, Tisch Scientific^®^) for VFA analysis. Concentration of VFA was analyzed using a high-performance liquid chromatograph (HPLC; Hitachi L2400, Tokyo, Japan)^45^.

The non-acidified samples were boiled at 100 °C for 10 min to denature enzymes and volatize VFA from the sample. Then, samples were centrifuged at 10,000×*g* for 15 min at 4 °C, and the supernatant transferred to a new microcentrifuge tube used for the determination of d-lactate, l-lactate, and total lactate concentrations. Lactate concentrations were analyzed by enzymatic reactions with a R-Biopharm kit^46^. Briefly, this was an enzymatic method divided into two steps, which were used for the determination of d- and l-lactate concentration, respectively. Lactate concentration was determined by using the d- and l-lactate dehydrogenase enzymes, and total lactate concentration was calculated from the sum of both lactate concentration.

The effluent contents and bacterial pellets were freeze-dried using a Labconco FreeZone 6 (Labconco Corporation, Kansas City, MO, USA). The dietary ingredients, background, effluent content, and bacterial pellets were analyzed for DM (method 934.01; AOAC, 1990), ash (method 924.05)^47^, and for total N and ^15^N enrichment [CHNS analyzer coupled with an isotope ratio mass spectrometer (dumas dry combustion method)^48^. The OM was considered as the difference between DM and ash contents. The CP concentration was calculated from the total N content (total N × 6.25; DM basis). Dietary ingredients, effluent contents, and bacterial pellets were analyzed for total glucose using an enzymatic-colorimetric method^49^ with the goal of determining the starch content of dietary nutrients and effluent contents, and the glycogen concentration in bacterial cells. Dietary ingredients and effluent contents were analyzed for NDF^25^ and subsequently analyzed for ADF^50^, both with an adaptation for the Ankom^200^ Fiber Analyzer (Ankom Technology, Macedon, NY). Dietary ingredients were also analyzed for ether extract (EE; method 920.85)^51^. The content of total digestible nutrients (TDN) was calculated using the following equations:

$${\text{TDN }} = {\text{ dCP }} + \, \left( {{\text{dEther}}\,{\text{extract }} \times { 2}.{25}} \right) \, + {\text{ dNDF }} + {\text{ dNSC}},\,{\text{which}}\,{\text{dNSC}}\,{\text{was}}\,{\text{equal}}\,{\text{to}}\,{\text{the}}\,{\text{digestible NFC}}{.}$$

### Ruminal pH, nutrient flows, and N metabolism calculations

Ruminal pH data was used to calculate the time in which the pH was within certain thresholds between feeding times [time in subacute ruminal acidosis (SARA; time in which pH was between 5.2 and 5.6); and time in which pH was below 5.2 (indicator of acute ruminal acidosis)]. Then, to quantify the major differences in pH, the area under the pH curve (area under the curve; AUC) was calculated for the aforementioned thresholds using the trapezoidal rule^20,52^, as follow:

$${\text{Time}}\,{\text{under}}\,{\text{a}}\,{\text{pH}}\,{\text{threshold}}, \, \% \, = { 1}00 \times \left( {{\text{time}}\,{\text{under}}\,{\text{threshold}},{\text{ h}}/{\text{day}}} \right)/{\text{total}}\,{\text{hours}}\,{\text{pH}}\,{\text{was}}\,{\text{measured}},\,{\text{h,}}$$

$${\text{AUC (pH }} \times {\text{ h}}/{\text{day )}} \, = {\text{ }}\sum [{\text{ (pH}}0 + {\text{ pH1 )}} \times \left( {{\text{t}}_{1} - {\text{ t}}0} \right)/2{\text{ ]}},$$

which pH_0_ and pH_1_ are two pH measurements in a pH interval t_0_ and t_1_, respectively.

For nutrient digestibility, because residues of nutrients from the non-acidotic diet (days 1–8) were still present on days 9–11, we reported true flow of dietary nutrients out of the fermenters, meaning the greater the nutrient flow value was the less digested it would have been. The true dietary nutrient flow was calculated as the total nutrient leaving the fermenter corrected by the concentration of that nutrient in the bacteria and the artificial saliva. The total N in the effluent content was partitioned in NH_3_–N and nonammonia N (NAN; undegraded feed N and bacterial N). Outflow of each of these fractions from fermentation were calculated following the equations described by Calsamiglia et al.^53^ and Bach and Stern^54^. Dietary N flow, bacterial efficiency, and the efficiency of N use (ENU) were calculated according to Calsamiglia et al.^53^. The calculations were as follow:

$${\text{NH}}_{{\text{3}}} {\text{ - N}} \, {\text{flow }}\left( {{\text{g}}/{\text{day}}} \right) \, = {\text{ NH}}_{{\text{3}}} {\text{ - N}} \, {\text{concentration}} \, {\text{in}} \, {\text{effluent}} \, {\text{containers }}\left( {{\text{mg}}/{\text{dL}}} \right) \, \times \, \left( {{\text{Liters}} \, {\text{of}} \, {\text{total}} \, {\text{effluent}} \, {\text{flow}}/100} \right),$$

$${\text{NAN}}\,{\text{flow }}\left( {{\text{g}}/{\text{day}}} \right) \, = {\text{ total}}\,{\text{N}}\,{\text{in}}\,{\text{effluent}}\,{\text{containers }}\left( {\text{g}} \right) \, - {\text{NH}}_{{\text{3}}} {\text{ - N}}\,{\text{in}}\,{\text{effluent}}\,{\text{containers }}\left( {\text{g}} \right),$$

$$\begin{aligned} {\text{Bacterial}}\,{\text{N}}\,{\text{flow }}\left( {{\text{g}}/{\text{day}}} \right) \, &= \, \left( {{\text{NAN}}\,{\text{flow }} \times \, \% \,{\text{atom}}\,{\text{excess}}\,{\text{of}}^{{{15}}} \,{\text{N}}\,{\text{in}}\,{\text{NAN}}\,{\text{flow}}} \right) \, \hfill \\& \quad \div \, \left( {\% \,{\text{atom}}\,{\text{excess}}\,{\text{of}}^{{{15}}} {\text{N in bacterial pellet}}} \right),{\text{ where }}\% {\text{ atom excess of}}^{{{15}}} {\text{N in NAN flow }} \hfill \\ & = \, \% {\text{ atom}}^{{{15}}} {\text{N in NAN flow }} - \, \% {\text{ atom}}^{{{15}}} {\text{N in background samples,}} \hfill \\ \end{aligned}$$

$${\text{Dietary}}\,{\text{N}}\,{\text{flow }}\left( {{\text{g}}/{\text{day}}} \right) \, = {\text{ NAN}}\,{\text{flow }} - {\text{ g}}\,{\text{of}}\,{\text{bacterial}}\,{\text{N}}\,{\text{in}}\,{\text{effluent,}}$$

$$\begin{aligned} & {\text{Bacterial}}\,{\text{efficiency }}\left( \% \right) \, = {\text{ bacterial}}\,{\text{N}}\,{\text{flow }}\left( {\text{g}} \right)/{\text{OM}}\,{\text{truly}}\,{\text{digested }}\left( {\text{g}} \right),\\ & {\begin{aligned} {\text{where}}\,{\text{OM}}\,{\text{truly}}\,{\text{digested }}\left( {\text{g}} \right) \, & = \, \left[ {{\text{g}}\,{\text{of}}\,{\text{OM}}\,{\text{intake }}{-}\left( {{\text{g}}\,{\text{of}}\,{\text{OM}}\,{\text{in}}\,{\text{effluent}}\,{\text{containers}}} \right.} \right. \\ & \quad \left. {\left. {{-}{\text{ g}}\,{\text{of}}\,{\text{OM}}\,{\text{in}}\,{\text{the}}\,{\text{artificial}}\,{\text{saliva }}{-}{\text{ g}}\,{\text{of}}\,{\text{OM}}\,{\text{in}}\,{\text{bacteria}}} \right)} \right], \end{aligned}} \end{aligned}$$

$${\text{ENU }}\left( \% \right) \, = \, \left( {{\text{bacterial}}\,{\text{N}}\,{\text{flow}}/{\text{g}}\,{\text{of}}\,{\text{available}}\,{\text{N}}} \right) \, \times { 1}00.$$

### Statistical analyses

Data were analyzed using the MIXED procedure of SAS as a replicated 4 × 4 Latin square design. The main model used for our data analyses was the following:

$${\text{Y}}_{{{\text{ijkl}}}} = \, \mu \, + {\text{ L}}_{{\text{i}}} + {\text{ P}}_{{\text{j}}} + {\text{ F}}\left( {\text{S}} \right)_{{{\text{ki}}}} + {\text{ TR}}_{{\text{l}}} + {\text{ D}}_{{\text{m}}} + {\text{ TR}} \times {\text{D}}_{{{\text{lm}}}} + {\text{ E}}_{{{\text{ijklm}}}} ,$$

which Y_ijkl_ is the response variable, µ is overall mean, L_i_ is the effect of Latin square (i = 1 or 2), P_j_ is the random effect of period (j = 1–4), F(S)_ki_ is the random effect of fermenter (F) within square (k = 1–4), TR_l_ is the effect of treatment, D_m_ is the effect of day, T × D_lm_ is the interaction between treatment and day, and E_ijkl_ is the residual error.

Volatile fatty acids and pH related variables calculated from the dynamics of pH, such as average pH, hours under certain thresholds, and AUC were analyzed using the following model:

$${\text{Y}}_{{{\text{ijkl}}}} = \, \mu \, + {\text{ Cov }} + {\text{ L}}_{{\text{i}}} + {\text{ P}}_{{\text{j}}} + {\text{ F}}\left( {\text{S}} \right)_{{{\text{ki}}}} + {\text{ TR}}_{{\text{l}}} + {\text{ D}}_{{\text{m}}} + {\text{ TR}} \times {\text{D}}_{{{\text{lm}}}} + {\text{ E}}_{{{\text{ijklm}}}} ,$$

_ijkl_ is the response variable, µ is overall mean, Cov is the covariate (data collected on day 7 of each period before applying treatments), L_i_ is the effect of Latin square (i = 1 or 2), P_*j*_ is the random effect of period (j = 1–4), F(S)_ki_ is the random effect of fermenter (F) within square (k = 1–4), TR_l_ is the effect of treatment, D_m_ is the effect of day, T × D_lm_ is the interaction between treatment and day, and E_ijkl_ is the residual error. Ruminal pH data, and NH_3_–N, d-lactate, l-lactate, and total lactate concentrations were analyzed overtime as repeated measures in a strip-plot arrangement of the variables DAY and TIME, further included in the model. The covariance structures tested in all models were: AR (1), ARH (1), CS, TOEP, TOEPH, UN, and VC; the structure with lowest AIC was chosen. Significance was declared at *P* ≤ 0.05 and trends at 0.05 < *P* ≤ 0.10. Tukey test was used to compare means whenever differences were observed.

## Supplementary Information

Supplementary Figures.

## Data Availability

All figures were produced by using the software DataGraph from Visual Data Tools (https://www.visualdatatools.com). All raw data are available by the authors upon request.
