# Supplementary material for: Megasphaera elsdenii and Saccharomyces Cerevisiae as direct fed microbials during an in vitro acute ruminal acidosis challenge
Source: Sci Rep. 2022 May 13;12:7978. doi: 10.1038/s41598-022-11959-2 (PMC9106753; doi:10.1038/s41598-022-11959-2)
Supplement: Supplementary file 1 — Supplementary Figures. [file 41598_2022_11959_MOESM1_ESM.docx]

**Figure Legends**

**Supplementary Figure S1.** Visualization of the dual-flow continuous culture fermenter used in the study when it was fed a non-acidotic diet (days 1-8). Fermenters were fed 107 g of DM per day equally divided into two meals (7:00 and 21:00 h). Artificial saliva was continuously infused in the fermenters as the buffer for ruminal fermentation. To simulate the urea recycling in the rumen, urea was added to the artificial saliva at a rate of 0.4 g/L. Because the dual-flow continuous culture system allows the passage rates of ruminal content out of fermenters to be pre-determined, the dilution rate was set at a rate of 10%/h while the passage rate of solids was set at a rate of 5%/h.

**Supplementary Figure S2.** Visualization of the dual-flow continuous culture fermenter used in the study during the challenge (days 9-11). Fermenters were fed 107 g of DM per day equally divided into two meals (7:00 and 21:00 h). Artificial saliva was continuously infused in the fermenters as the buffer for ruminal fermentation. To simulate the urea recycling in the rumen, urea was added to the artificial saliva at a rate of 0.4 g/L. Because the dual-flow continuous culture system allows the passage rates of ruminal content out of fermenters to be pre-determined, the dilution rate was set at a rate of 10%/h while the passage rate of solids was set at a rate of 5%/h.

**
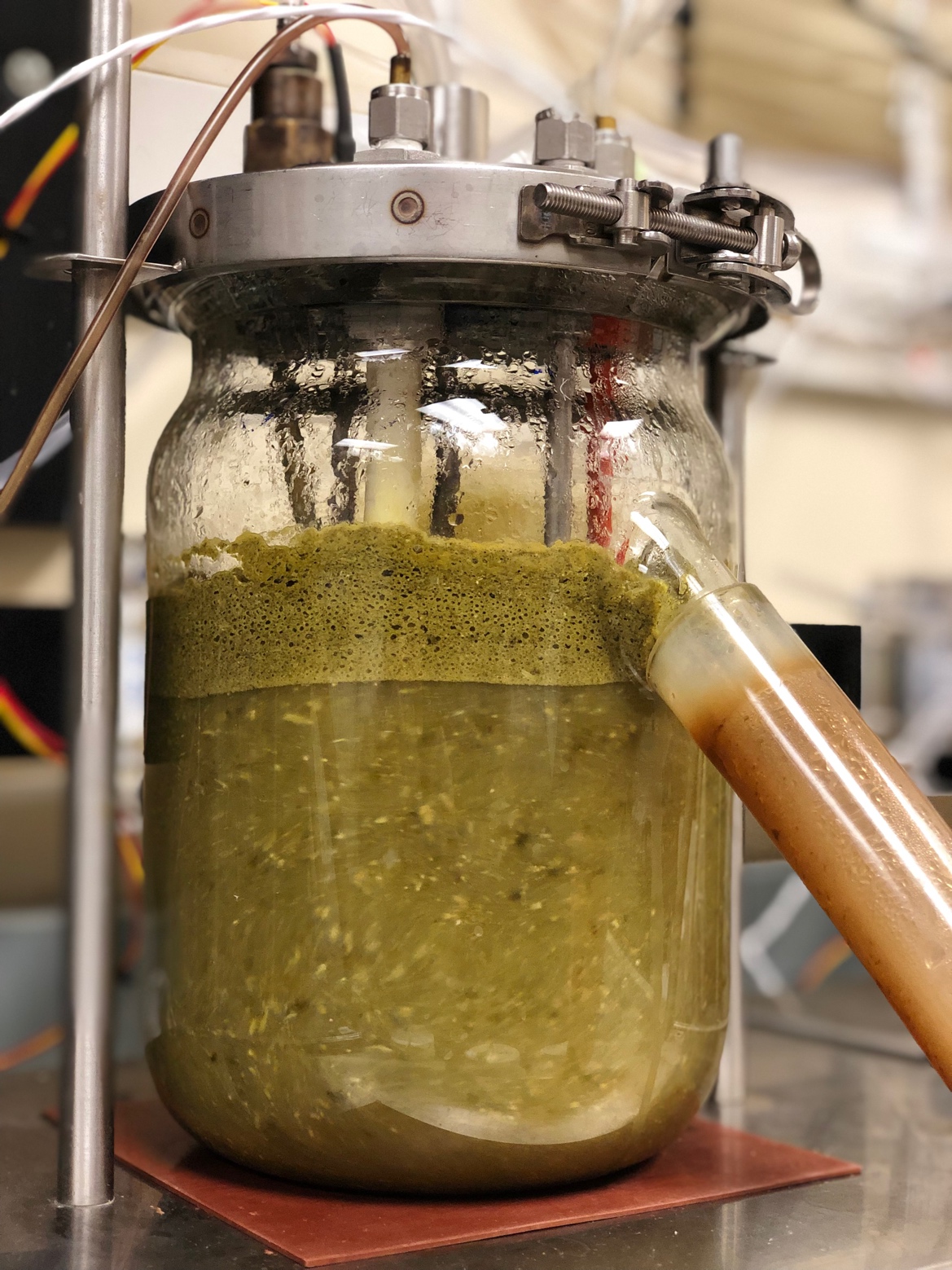
**

**Supplementary Figure S1**

**
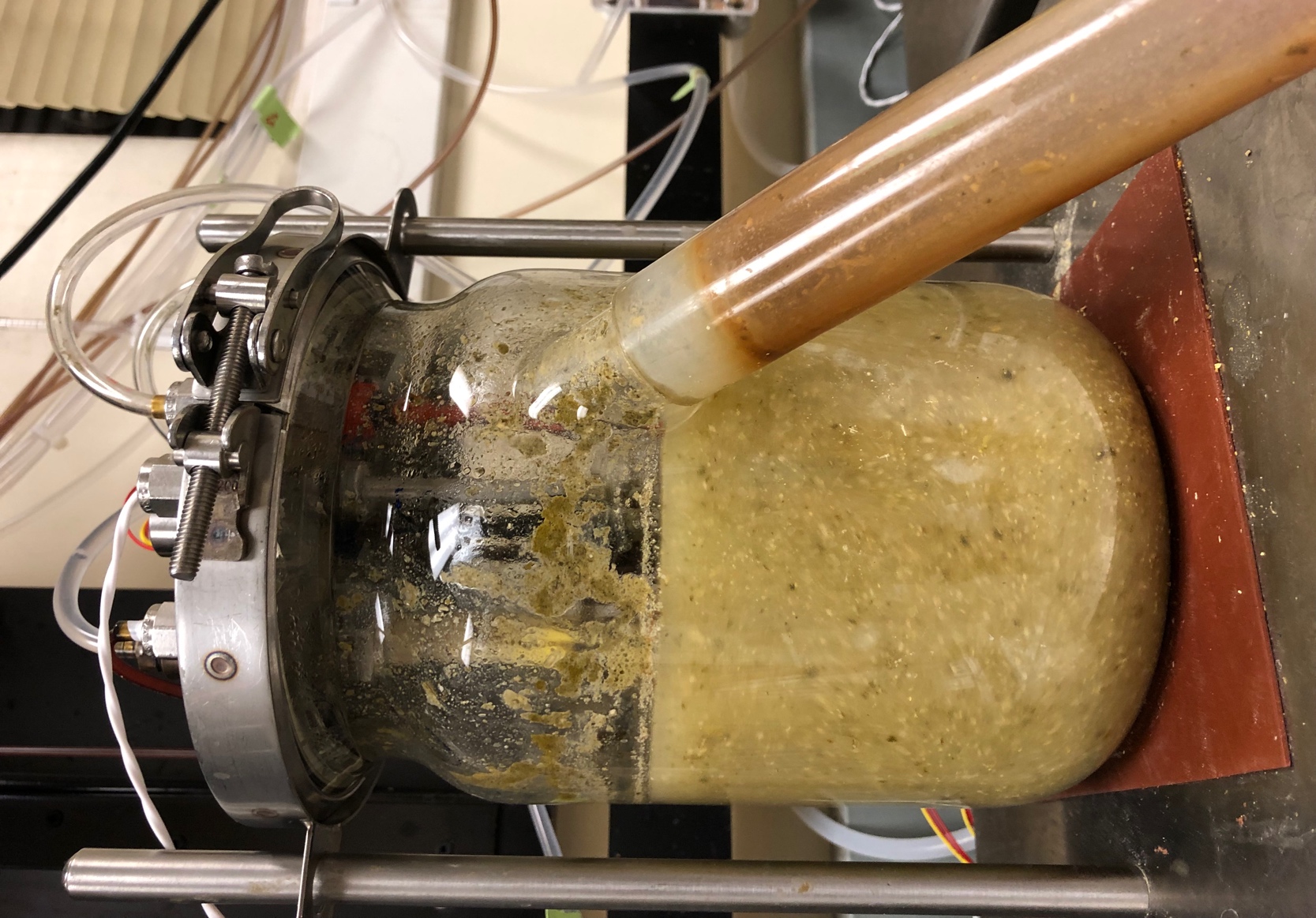
**

**Supplementary Figure S2**
